# Amplifying Their Voice: Inclusive Healthcare Provider Perspectives to Improve Advancement, Resilience, and Retention

**DOI:** 10.7759/cureus.66028

**Published:** 2024-08-02

**Authors:** Cora Breuner, Emily Moore, Elaine Walsh, Stephanie Hilman, Julia Mitzel, Anita Thomas, Leslie Walker-Harding

**Affiliations:** 1 Adolescent Medicine, Seattle Children's Hospital, Seattle, USA; 2 Pediatric Cardiology, Seattle Children's Hospital, Seattle, USA; 3 Pediatrics, Seattle Children's Hospital, Seattle, USA; 4 Pediatrics, Impact4Health, Seattle, USA

**Keywords:** inclusion and diversity, diverse research questions, job satisfaction, resilience, retention, gender bias, nonbinary, advanced practice practitioners, inclusion, equity

## Abstract

Background and objectives: Addressing the issues of workplace advancement, resilience, and retention within medicine is crucial for creating a culture of equity, respect, and inclusivity especially towards women and nonbinary (WNB) providers including advanced practice providers (APPs), most notably those from marginalized groups. This also directly impacts healthcare quality, patient outcomes, and overall patient and employee satisfaction. The purpose of this study was to amplify the voices on challenges faced by WNB providers within a pediatric academic healthcare organization, to rank workplace interventions addressing advancement, resilience, and retention highlighting urgency towards addressing these issues, and, lastly, to provide suggestions on how to improve inclusivity.

Methods: Participants were self-identified WNB providers employed by a pediatric healthcare organization and its affiliated medical university. An eligibility screener was completed by 150 qualified respondents, and 40 WNBs actually participated in study interviews. Interviews were conducted using a semi-structured interview guide to rank interventions targeted at improving equity, with time allotted for interviewees to discuss their personal lives and how individual circumstances impacted their professional experiences.

Results: WNB providers called for efficient workflows and reducing uncompensated job demands. Support for family responsibilities, flexible financial/compensation models, and improved job resources all were endorsed similarly. Participants ranked direct supervisor and leader support substantially lower than other interventions.

Conclusions: Career mentorship and academic support for WNB individuals are recognized interventions for advancement and retention but were not ranked as top priorities. Respondents focused on personal supports as they relate to family, job resources, and flexible compensation models. Future studies should focus on implementing realistic expectations and structures that support whole lives including professional ambitions, time with family, personal pursuits, and self-care.

## Introduction

In 2018, the National Academies reported gender bias within the myriad macro- and micro-aggressive behaviors (gaps in academic ranking, salary, grant funding, and overall hostility) that can make women feel "less than" in the workplace [1]. It has been documented that such discrimination correlates with burnout and attrition [2,3].

The woman provider exodus from the health workforce is counterproductive to efforts at improving healthcare. Studies have shown that patients have better outcomes with women healthcare providers [4-6], yet despite this, a large number of women are reducing hours or leaving the workforce and may miss opportunities for potential leadership [7,8].

Discrimination impacting women and people of color poses a significant threat to workplace inclusion [9]. Addressing this is crucial for creating a workplace culture of equity, respect, and inclusivity. Overlooking the needs of women and nonbinary (WNB) medical doctors (MDs)/doctors of osteopathy (DOs)/behavioral health specialists and advanced practice providers (APPs) (referred to collectively as WNB providers), especially those from the marginalized cultural groups, directly impacts the quality of and relates to poorer patient outcomes [10], reduced patient satisfaction [11], and financial impacts of replacing a provider [12]. Research shows that retaining WNB providers (i.e., providers who do not identify as male) improves clinical decision-making, increases patient satisfaction, enhances learner satisfaction, and fosters diverse research questions [13,14]. The benefits for workers include a sense of purpose, sustained professional balance, increased job satisfaction, and decreased turnover [15].

A 2022 survey study focusing on pediatric faculty documented that discriminatory treatment was more likely to be experienced by women versus men at least once a year (51% vs. 4%); encountering sexist remarks, unwanted attention, or advances was disproportionately experienced by women (24% vs. 15%) [16]. Participants reported that discriminatory experiences negatively impacted their confidence and career advancement. Unfair gender treatment was especially noted by those who identified as Asian, Black/African American, Hispanic/Latino, and mixed race or other race(s). Based on this study, interventions are needed to stem these practices and help retain people with different backgrounds.

The purpose of this study was to amplify voices on the challenges faced by WNB providers with an emphasis on diversity within a pediatric academic healthcare organization and to rank interventions known to address advancement, resilience, and retention [17].

We obtained qualitative data to rank a list of interventions the WNB population found most necessary for workplace satisfaction and retention. This study was approved by the Institutional Review Board of Seattle Children's Hospital (approval number: STUDY00003780).

## Materials and methods

Design

This study used a qualitative descriptive design to systematically process data collected through interviews, staying close to the surface of the content, meaning the data was interpreted as it was received, looking for common themes [18].

Sample and setting

An email was sent from medical staff services to the pediatric academic healthcare organization's providers explaining the study. Inclusion criteria were self-identifying as WNB individuals and employment by the pediatric academic healthcare organization or the affiliated medical university. Participants were WNB physicians, psychologists, and APPs. An APP was either a nurse practitioner or a physician assistant who performs medical activities. This healthcare organization is in Washington state, which is a full practice authority state for nurse practitioners, meaning a nurse practitioner is an independent provider in which no other healthcare professional has authority over their ability to practice. Physician assistants practicing in Washington state require a practice agreement with a physician, but the majority of their practice is independent [19]. Given the clear overlap within these provider roles, a heterogenous sample was developed. An eligibility screener was completed by 150 respondents, from which a sample of 40 was purposefully selected to narrow down a diverse sample of participants to represent the providers within the organization. We used both purposive and convenience sampling techniques.

Participants completed a 60-minute 1:1 interview recorded over secure video conferencing by an outside agency. The same individual in the agency completed all 40 interviews. No participants declined the study or recording. Interviews were conducted between June 24, 2022, and January 6, 2023. Of note, the interviewer identified as female. 

Procedures and data analysis

Interviews were performed using a semi-structured interview guide. Prior to interviews, participants identified changes that would improve the work environment; we asked them to think broadly about actions that would help medicine amplify the voice and needs of WNB providers.

Participants were asked to review five interventions for improving provider well-being (see Table 1) and rate the relative importance of each. Next, they selected their top 3 interventions. Responses were included in the first interview question, which asked them to reflect on the interventions, how they decided on their top 3, and to identify concrete steps to make changes happen. Data was aggregated to assure participants could not be identified.

**Table 1:** List of intervention choices and percentage endorsing as priority Note: Participants endorsed three items, percentage totals >100% EMR: electronic medical record

Intervention	Percent prioritizing item in top 3	Specific examples/suggestions	
Create efficient workflows and reduce uncompensated job demands	35%	Side-by-side support for EMR documentation. Process improvement for clinic flow, including multiple check-ins on what is and is not working. Flexibility for provider schedules. Ability to build specialized teams and focus on retention. Establishing team communication norms, especially when out of the office and after hours. Train for clinical administrative responsibilities so it feels less of a burden	
Support family responsibilities	23%	Support options to grow a family, including fertility/egg-freezing procedures. Post-partum care should include lactation access and support, sleep education, and routine and emergency childcare. Create an infrastructure that allows for flexible schedules and coverage for illnesses and daily life events; allow for acceptable standards around "off time." Recognize individuals in the workplace, support their full lives, honor their role as caregivers at home and at work, and respect the importance of families and parents' presence in their children's lives, balancing dual roles as caregiver and provider	
Develop flexible financial and compensation models	19%	Increased salaries through raising benchmarks. Recognize additional responsibilities with compensation, building equitable advancement opportunities beyond academia. Evolve promotion practices to include leadership and training	
Improve job resources and equitable access to them including emotional and social support	17%	Invest in personnel for more equitable distribution of labor throughout the clinical system. Increase access to skilled administrative and clinical supports including interpreter services and more awareness of and access to leadership development opportunities. Mental health support	
Receive direct supervisor/leader support	7%	Train on topics related to bias, leadership skills, human resources, and strategic planning. Hire more women into leadership roles, which would not only address gender biases but increase visibility and presence of women in leadership. Clear guidelines to address roles and responsibilities and respect for people's boundaries within these roles eliminating penalties or retaliation for those who enforce them	

The primary focus of the interviews was to rank a pre-established list of interventions, with time allowed to discuss their personal lives and how individual circumstances impacted professional experiences. The interviewer captured comprehensive notes during the interview. To confirm validity and check for saturation of findings, five study team members verified transcripts by checking them against the recorded interviews. Saturation was achieved prior to the completion of 40 interviews.

Comments were grouped into similar themes and categories under each intervention. Interview data were analyzed by five study team members to identify common themes. We used qualitative content analysis, a dynamic form of analysis of verbal and visual data that focuses on summarizing informational contents of data [20,21]. We organized codes into themes. Themes were verified independently by team members who later met and discussed discrepancies until consensus was reached and dependability of findings [22] was confirmed. The final stage involved reviewing summary statements and assuring the accuracy of identified themes. 

## Results

The participants' age range was 29-72 years (mean 43.5). The participants reflected a diverse group of providers. Two participants were deaf or hard of hearing; for these, subtitles and interpreters were used within their interviews. Thirty-eight participants identified as women, and two participants were either genderqueer/nonbinary or transgender. See Table 2 for details.

**Table 2:** Study participants (n=40)

Characteristic	N	(%)
Race/ethnicity		
Asian	4	(10)
Black	4	(10)
Latinx/Hispanic	4	(10)
Mixed race (combinations of American Indian/Alaska Native, Asian, Black, non-Hispanic Caucasian)	6	(15)
Non-Hispanic White	22	(55)

Participants were asked to review five interventions for improving provider well-being. *Create efficient workflows and reduce uncompensated job demands* consisted of streamlining clinical responsibilities to remove tasks (extra meetings, committee work, etc.) without remuneration.* Support family responsibilities *included standardized, expanded, and even encouraged, family leave; cover time for non-birthing parents, adoption, foster care, and illnesses. *Develop flexible financial and compensation models* consisted of improving transparency of equitable pay and the practice of asking for it both at the time of interviewing and while negotiating salaries. *Improve job resources and equitable access to them including emotional and social support *was related to improvements to necessary breaks and the accumulation of time off. Finally, *Receive direct supervisor/leader support *included develop programs to retain marginalized individuals. 

WNB providers ranked efficient workflows and reducing uncompensated job demands first. Support for family responsibilities, flexible financial and compensation models, and improved job resources were endorsed similarly and differed by only a few percentage points. Finally, at 10% below the fourth ranked intervention was direct supervisor and leader support. 

## Discussion

The following discussion is not new to healthcare yet leaves many surprised. Despite previously published literature showing that women and minorities feel under-supported when trying to balance family and career responsibilities, this study highlights these issues continue with ongoing challenges. Additionally, it shows there is a need for ongoing change within the workplace. Many participants voiced frustrations related to feeling under-supported when it came to balancing their dual role as caregivers as pediatric healthcare professionals and in their personal lives. Many participants felt laden with administrative tasks, penalized for expanding their families, burdened by inequitable leave policies, encumbered with making accommodations for childcare, and scrambling for coverage when children were ill. These challenges extended into their homes, where many carried extensive household and childcare duties.

This study suggests healthcare organizations should focus on addressing these ranked interventions with a focus on efficient workflows, supporting families, flexible financial and compensation models, equitable access to support resources, and direct access to leader support. Compensation transparency, parity, and hierarchy flattening can address several of the above interventions, as can considering divisional/departmental differences in job resources and workflows. Recognizing different familial responsibilities and providing flexibility for childcare and household duties may also be an effective method of improving WNB provider job satisfaction and retention. The inclusion of WNB and marginalized providers in decision-making is crucial.

While it is well documented that support from leaders and supervisors is critical to job satisfaction and career success [23] and that men providers feel more supported than women providers [3], participants in this study did not rank this as a top priority. One reason for this could be citizen tax and woman tax [24]; when one is in survival mode, it can be difficult to see possibilities for advancement. Participants might also fail to see the potential for supervisor support when organization-level decisions seem to be less than supportive, they do not have time to interact with supervisors, or they may feel that their supervisors are not supportive.

A culture change that maximizes the potential of WNB is needed for more diverse and inclusive leadership [25].

The medical profession requires a major transformation with realistic expectations and structures that support whole lives including professional ambitions, time with family, and self-care. With time and change, there will be universal advantages [26-29]. A cataclysmic shift in support would benefit everyone involved in the healthcare environment, not solely WNB and marginalized providers. The future healthcare system must be more expansive, inclusive, and regenerative.

Future research directions include investigating fiscal components related to the interventions. Inclusive research is needed to understand the role racism and ableism play in marginalized WNB retention in the healthcare workforce.

Limitations

Participation was based on an invitation from medical staff services, which might have been perceived as a risk to confidentiality. Also, this study was limited to our organization. Third, our interviewer transcribed interviews without sending them to a third party for verbatim transcriptions. Interviews were conducted over Zoom, and while this is especially useful for referencing later and transcribing into written words, non-verbal communication is easily lost in translation. Finally, the study sample had a limited number of subjects. Initially, there were 150 interested respondents which were purposefully narrowed down to a diverse sample of participants to represent the providers within this organization. Additionally, all who identified as nonbinary were included. Despite limitations, the information presented adds to the discussion about the experiences of WNB providers and ways to retain them in the workforce.

## Conclusions

While several studies have examined retention and treatment of women in the workplace, this study is the first that we know of to examine and prioritize interventions in a population of WNB healthcare providers, with an intentional focus on a sample that differed in race, age, clinical specialty, and household composition. Findings underscore the urgency to demonstrate respect for this population of clinicians by providing the support needed to stay in practice. Indeed, mentorship, sponsorship, and support for WNB individuals throughout their careers are critical to ensure that they achieve academic milestones for career advancement and retention; however, this was not a priority in our interviews. Interventions should be tailored to the backgrounds and needs of providers. A new paradigm is necessary for WNB providers to attain leadership positions based on their skills and academic achievements.
